# Internalizing Profiles of Homeless Adults: Investigating Links Between Perceived Ostracism and Need-Threat

**DOI:** 10.3389/fpsyg.2019.00350

**Published:** 2019-02-20

**Authors:** Nejra Van Zalk, Rebecca Smith

**Affiliations:** ^1^Dyson School of Design Engineering, Imperial College London, London, United Kingdom; ^2^Department of Psychology, Social Work and Counselling, University of Greenwich, London, United Kingdom

**Keywords:** homelessness, internalizing psychopathology, ostracism, need-threat, person-oriented approach

## Abstract

Social ostracism among the homeless is a prevailing problem, yet few studies have focused on whether internalizing psychopathology moderates the links between feeling ostracized and perceiving threats to fundamental human needs. This study used a person-oriented approach to identify commonly occurring profiles of internalizing psychopathology characterized by symptoms of social anxiety, generalized anxiety, and depression (Low, Medium, and High Internalizers) among homeless participants residing in London, United Kingdom (*N* = 114; age range = 18–74; *M*_age_ = 46; 25% women). Data on perceived ostracism (feeling ignored and daily discrimination) and need-threat (belonging, self-esteem, meaningful existence, and feelings of control) was also collected. Controlling for the effects of age, living arrangement, gender, and time being homeless, feeling ignored was a significant predictor of need-threat, whereas daily discrimination was not. One significant interaction on the links between daily discrimination and need-threat emerged between Low and Medium Internalizers. For Medium Internalizers, high levels of daily discrimination were associated with high levels of need-threat. The effect was similar for High Internalizers and the opposite for Low Internalizers, though it was not significant within those groups. Taken together, these results indicate that differences in patterns of internalizing psychopathology should be taken into account when attempting to make homeless individuals feel more included in their surroundings.

## Introduction

Homeless adults are one of the most vulnerable groups in societies around the world today, as homelessness can help exacerbate poor mental health (Fitzpatrick-Lewis et al., 2011). Despite varieties in definitions across countries, a person is typically identified as homeless if they sleep rough (i.e., sleep out in the open or on the streets), use temporary shelters/hostels, or live in various types of transitional accommodations (Fazel et al., 2014). Though there is likely a complex reciprocal relationship between poverty and homelessness, as poverty is implicated as both a precursor and consequence of being homeless (see Bramley and Fitzpatrick, 2017 for an extensive discussion), the focus of the current study is on individuals who self-identify as homeless regardless of poverty levels. More than 400.000 individuals are estimated to be homeless on any one night in the EU, with 600.000 in the United States (Fazel et al., 2014). A large annual survey examining various characteristics of the homeless population in England reported that 28% used supported sleeping locations, 41% reported sleeping in hostels, 9% were rough-sleeping, 9% were sofa surfing/squatting, 6% were in emergency/temporary sleeping arrangements, 5% reported having their own home, and 1% reported having other arrangements (Harris, 2017). Importantly, the consequences of being homeless are many and far-reaching.

One common experience of homelessness is ostracism (Johnstone et al., 2015; Carpenter-Song et al., 2016). Defined as “*ignoring and excluding individuals or groups by individuals or groups*” (Williams, 2007, p. 427), ostracism is similar to concepts such as rejection (declaring explicitly that an individual is not wanted) and social exclusion (being kept apart from others; Williams, 2007). Because of their similarity in terms of outcomes (Williams, 2007; Smart Richman and Leary, 2009), all of these terms were included in the current literature review. Ostracism ranges from subtle signs of dismissal such as ignoring eye-contact, to more openly sanctioned forms used by both individuals and institutions such as discrimination (Williams and Zadro, 2001; Williams, 2007). Typical homelessness characteristics (including for example lack of housing or lack of social support) are also considered components of social exclusion – a fact which has likely resulted in a dearth of research on homeless people’s social exclusion in particular (Van Straaten et al., 2018). Moreover, social exclusion could be a cause as well as a result of homelessness (Clapham, 2003). It has been argued that homeless people largely feel socially excluded in Western societies, where individualism and self-sufficiency are highly valued (Carpenter-Song et al., 2016). Belcher and DeForge (2012) maintain that stigmatization of the homeless is inevitable within a capitalist society which is inherently unequal, and justification of this inequality encourages people to place the blame on the individuals who “fail.” Homeless individuals are ostracized for a variety of reasons, including others viewing their stigmatized identity as partially controllable (e.g., as resulting from drug addiction; Johnstone et al., 2015) – and negative treatment on group level is perceived as more legitimate if directed toward individuals whose stigma is believed to be under their own control (Weiner et al., 1988). In a recent survey of public attitudes to homelessness in the United Kingdom (O’Neil et al., 2017), the authors found that people were inclined to see the causes of homelessness in terms of bad decisions, failures, or misfortunes on behalf of the homeless person. Indeed, simply labeling a target person as homeless has been shown to result in negative evaluations by others (Phelan et al., 1997). There is also strong evidence for a high level of dehumanization toward homeless individuals in particular, with feelings of disgust activated in brains of participants viewing photographs of homeless people (Harris and Fiske, 2006). In addition, homeless individuals report high levels of group-based discrimination, which likely hinders them from connecting to others in turn (Johnstone et al., 2015). Thus, homeless individuals are particularly vulnerable to ostracism, rejection, and social exclusion by others.

According to Williams (2009), the rapid detection of even the most minimal form of ostracism happens reflexively in the form of pain (such as sadness or anger) as well as threats to fundamental needs. This early detection of ostracism is likely of evolutionary value because rejection by a group has survival implications (Williams, 2009). The pain is followed by a reflective phase, whereby people question, appraise and attribute motives or relevance to the ostracism situation, which in turn leads to need fortification (either via attempting to change/conform, or via getting attention/provoking). The final, long-term effect of persistent ostracism is resignation, whereby the individual hasn’t been able to replenish or fortify their needs, and the end result could be a wide array of issues such as depression, learned helplessness, reduced psychological resilience, unworthiness, and alienation (Williams, 2009). A wealth of evidence, particularly from short-term lab manipulations of ostracism, shows that it depletes fundamental needs (see Hartgerink et al., 2015 for a review), and is experienced in a manor akin to physical pain (as demonstrated using fMRI; Eisenberger et al., 2003). Because homeless individuals are particularly vulnerable to rejection and ostracism, they represent a specific group of interest in order to test elements of Williams (2007) theory linking ostracism to need-threat. As has been pointed out, more research on ostracism among the homeless is required (Williams, 2009), and though one would expect a specific impact of social ostracism on need-threat in this population, this needs to be explored further. Although Williams (2009) proposed chain of events following ostracism has intricate temporal implications, in this study we are focusing on the links between feeling ostracized and threatened needs in particular.

Homeless individuals also commonly exhibit other vulnerabilities that make them particularly sensitive to the effects of social ostracism, such as internalizing problems. Adult internalizing psychopathology, which is characterized by inner distress (Yoder et al., 2008), can be understood via a hierarchical factor model similar to internalizing models in childhood. Internalizing represents one of two broad correlated hierarchies (Externalizing being the other) that is characterized by a spectrum of phenomena such as depression, anxiety, and various phobias (Krueger and Markon, 2006a,b; Hankin et al., 2016). The link between homelessness and internalizing psychopathology has been well-documented, as homeless individuals commonly report high symptoms of depression and anxiety (e.g., Edens et al., 2011; Fitzpatrick et al., 2012, 2015; Cruwys et al., 2014; Fazel et al., 2014; Fitzpatrick, 2017). Depressive symptoms, generalized anxiety, and social anxiety also commonly co-occur in adolescence (e.g., Epkins and Heckler, 2011; Waszczuk et al., 2016) and adulthood (e.g., Kessler et al., 2005) in the general population, but research on their co-occurrence in the homeless population is sparse (Hodgson et al., 2013). Importantly, internalizing psychopathology could either be a vulnerability/risk factor for homelessness, a consequence of being homeless, or likely both. For instance, depression and low self-esteem were found to moderate the reactions to being socially excluded in a sample of female undergraduate students (Nezlek et al., 1997), and similar links might be expected for homeless individuals. Socially anxious adults report high levels of threat to their primary needs and slow recovery from ostracism experiences (Zadro et al., 2006) – though this hasn’t been examined in the homeless population. In addition, recent and past stressors such as significant life events paired with traumatic childhood experiences likely increase homeless individuals’ internalizing symptoms (Montgomery et al., 2013; Fitzpatrick, 2017). Evidence from the general population suggests that internalizing psychopathology exacerbates how people respond to difficult situations (Keltner and Kring, 1998), and rumination – a key component of internalizing psychopathology – slows ostracism recovery (Wesselmann et al., 2013). Recent empirical work on the links between homelessness and social exclusion showed that those who report less social relatedness have the highest increased psychological distress (Van Straaten et al., 2018). In this paper, we assume that internalizing psychopathology is already present on an individual level, and thus either buffers or strengthens (i.e., moderates) the link between ostracism and threats to fundamental needs.

The majority of existing studies examining mental health in homeless populations employ variable-centered approaches, but there are advantages to using an alternative perspective such as a person-oriented approach. Indeed, “*modeling/description of variables over individuals can be very difficult to translate into properties characterizing single individuals because the information provided by the statistical method is variable oriented, not individual oriented*” (Bergman and Magnusson, 1997, p. 2). Even though the person-oriented approach also relies on variables, these are considered components of a particular pattern under analysis and interpreted in relation to other variables under consideration. One typical approach to studying psychopathology is categorizing continuous variables (in itself problematic; see Rucker et al., 2015), thereby employing extreme or clinical levels of for example depressive symptoms based on arbitrary cut-offs that do not necessarily reflect the wide dimensionality of problems (Harvey et al., 2004). A person-oriented approach instead emphasizes a *holistic-interactionis*t view of human development by identifying profiles or patterns of data as a useful methodological tool for the study of interindividual differences (Bergman et al., 2003). The individual is seen as an organized whole, where at each level the individual’s totality receives its characteristic features from the interaction between involved elements (Bergman et al., 2003). Thus, people are expected to exhibit different patterns of mental, biological, and behavioral factors operating at all levels of individual functioning. To understand such patterns, identification of heterogeneous, naturally occurring profiles of individuals within a group is necessary. To our knowledge, only one study has directly sought to identify profiles of homeless individuals based on mental health patterns. Three profiles of homeless young people were identified: those with minimal mental health issues; those with mood, substance, and conduct disorder problems, and those with post-traumatic stress disorder, mood, and anxiety issues (Hodgson et al., 2015). Notably, the profiles differed on various characteristics such as mental health status, service use and suicide risk on follow-up. More research on profiles of internalizing psychopathology among homeless adults is thus still required. To our knowledge, whether homeless adults with different profiles of internalizing psychopathology vary in terms of perceived ostracism and experiences of need-threat is currently unexplored.

Several important factors might interfere with the aforementioned processes. Length of homelessness plays a role in the development of mental health problems (Fitzpatrick, 2017), as newly homeless youths have reported relatively low rates of mental health problems in some studies (e.g., Rosenthal et al., 2007). Age itself might also be an important factor, on the other hand, as younger homeless individuals have demonstrated more severe mental health and substance abuse problems compared to older individuals in some studies (Gordon et al., 2012), but not in others (up to 78 years; Tompsett et al., 2009). In addition, an individual sleeping rough likely feels more excluded compared to a person sleeping in a hostel (Clapham, 2008), whereas perceptions of the social environment (e.g., being in a homeless shelter) are associated with poorer mental health (Beharie et al., 2015). Finally, homeless women report higher levels of psychopathology compared to homeless men, and are more likely to internalize rather than externalize their traumatic experiences (Jainchill et al., 2000). For these reasons, controlling for length of homelessness, age, living arrangement, and gender would be of importance in subsequent analyses.

In this study, we recruited an opportunity sample of homeless adults with multiple complex needs (*N* = 114; age range = 18–74; *M*_age_ = 46; 25% women) from a number of hostels in London. We collected self-reports on symptoms of internalizing psychopathology (generalized anxiety, social anxiety, and depressive symptoms), perceived social ostracism (feeling ignored and daily discrimination) and need-threat (belonging, self-esteem, meaningful existence, and control) using a survey design. As mentioned previously, the links between ostracism and need-threat have a presumed temporal sequence, whereby ostracism affects need-threat in turn (Williams, 2009). Because our data is based on cross-sectional self-reports (i.e., perceptions), temporal aspects cannot be tested – thus we assume this particular temporal succession based on previous theory and empirical research. In addition, even though internalizing psychopathology could precede as well as result from these processes, we focus on its moderating effects by postulating that the participants are already exhibiting various levels of internalizing stressors. That is, the temporal antecedence of internalizing psychopathology is assumed rather than tested. To identify profiles of internalizing symptoms among the participants, we conducted cluster analysis with generalized anxiety, depressive symptoms and social anxiety as outcomes. We expected to identify profiles of homeless individuals with varying degrees of symptoms of internalizing psychopathology (e.g., high vs. low internalizing). Controlling for the effects of age, gender, current living arrangement, and time spent being homeless, we also expected that internalizing would interact with perceptions of ostracism in predicting threats to primary needs. That is, those with high levels of internalizing and ostracism were expected to exhibit the highest levels of need-threat compared to those with lower levels of internalizing.

## Materials and Methods

Participants were 114 adults (age range = 18–74; *M*_age_ = 46; 25% women) associated with the Single Homelessness Project (SHP), a 40-year old London-based charity focused on preventing homelessness and helping vulnerable and socially excluded people. Our main point of contact was Fulfilling Lives Camden & Islington (FLIC), a Big Lottery-funded charity organization within SHP, whose chief focus is supporting homeless people with multiple complex needs (i.e., individuals who exhibit manifold problems such as drug and alcohol use as well as mental health problems). As this was an opportunity sample, due to the particular vulnerability of and the difficulty in engaging these participants, we aimed to collect data from the largest possible number of participants within the time scope of the study (thus no *a priori* power analyses were conducted). In addition, the particular vulnerability of participants with multiple complex needs played a role in the choice of measures for the study. For example, we selected measures that were deliberately short and easy to comprehend so as to ensure a higher level of willingness to participate (for example, social ostracism was measured with the Ostracism Experience Scale for Adolescents, which was chosen for its brevity).

Some participants were housed/sheltered via SHP, whereas others were homeless or sleeping rough but partaking in SHP activities. FLIC provided contact information, and the data were collected at day centers (76%), hostels (15%), tenancies (1%), or via the FLIC office phone (8%), with no differences for any of the main study variables regardless of where the data collection took place (*p*’s > 0.10). Data missingness was low (0.9% for some of the study variables), and Little’s MCAR test showed that the data were missing completely at random (*Sig*. = 0.997). Sixty percent of the sample were White, 16% were Black, 12% were Asian, 9% were Mixed Race, and 3% identified as Other. Twenty-five percent reported living in a tenancy, 35% lived in a hostel, 1% lived in a shelter, 30% lived on the street, and 9% reported having other living arrangements. Thirty-two percent had been homeless up to 1 year, 22% had been homeless 2–3 years, 16% had been homeless 3–5 years, and 30% had been homeless 5 years or longer.

### Procedure

Participants were recruited during daytime. They were informed about the study and given a consent form to sign before participating. They were guaranteed full anonymity and given the option to withdraw their data by creating a 4-digit code only they would remember, which was linked to their questionnaire (none chose to do so). They were also told that they didn’t have to answer any questions they felt uncomfortable with. All of the participants were debriefed following the data collection.

The questionnaires were administered by a trained Research Assistant who was accompanied into the shelters by in-house staff. No staff were present during the data collection, though staff were available on-site. A few individuals were incapable of filling out the questionnaires themselves due to problems such as poor reading skills or anxiety, in which case the Research Assistant read the questions and response items, and these were filled out according to the participant instructions. The majority of the participants filled out the questionnaires entirely on their own, however. No participant was paid for taking part in the study, but they received biscuits, coffee, and a pair of socks if they chose to take them. The same incentives were offered to all participants, whether or not they chose to participate or to finish their participation. The procedures and measures were approved by the University’s Ethics Review Board.

### Measures

#### Internalizing Indices

We used three indicators of internalizing psychopathology: symptoms of generalized anxiety, depression, and social anxiety.

##### Generalized anxiety

Symptoms of generalized anxiety were measured with the shortened version of the Spielberger State-Trait Anxiety Inventory (STAI; Marteau and Bekker, 1992). The scale includes six items, where the participants were asked how they’ve felt during the past week. Examples include “upset,” “tense,” and “content” (reversed). Response items ranged from *Not at all* (1), *Somewhat* (2), *Moderately* (3), to *Very much* (4). The scale has demonstrated acceptable reliability and validity in previous studies (Marteau and Bekker, 1992). The Cronbach’s α reliability in this study was 0.74.

##### Depressive symptoms

We measured depressive symptoms such as worry, sadness, hopelessness, lethargy, and poor appetite with the 10-item shortened version (Cheng et al., 2006) of the widely used Center for Epidemiologic Studies Depression Scale (CES-D; Radloff, 1977). The shortened version includes 10 questions, and has previously demonstrated good factorial validity (Cheng et al., 2006). Examples of statements were: “During the past week I felt down and unhappy,” “During the past week I felt lonely, like I didn’t have any friends,” and “During the past week I felt like I was too tired to do things.” The response items ranged from *Not at all* (1), *Very seldom* (2), *Now and then* (3), to *Often* (4). The Cronbach’s α reliability for this measure was 0.82.

##### Social anxiety

Social anxiety was measured with 14 questions about fears in different social situations using the Social Phobia Screening Questionnaire (SPSQ; Furmark et al., 1999). The instrument comprises two different parts and correlates highly with other indicators of social anxiety disorder (Furmark et al., 1999). We only used the first part of the scale pertaining to social situations that tend to elicit symptoms of social anxiety, such as “speaking in front of a group of people,” “attending a party (or social gathering),” and “expressing opinions in front of others.” This approach has been used in previous research to measure subclinical levels of social anxiety (e.g., Van Zalk and Tillfors, 2017). The response items ranged on a 5-point scale from *No distress* (0), (1), (2), (3), to *Severe distress* (4). The Cronbach’s α reliability was 0.89.

#### Indices of Ostracism

We used self-reports of feeling ignored and feeling discriminated against as indicators of social ostracism.

##### Feeling ignored

Social ostracism was measured with the Ostracism Experience Scale for Adolescents (OES-A; Gilman et al., 2013). The original scale consists of two subscales gauging feeling ignored and feeling excluded, respectively. For this study, we only used the feeling ignored subscale, which comprises five items about how one is generally treated by other people. This subscale has shown good predictive validity of measures of psychological distress and well-being (Gilman et al., 2013). Examples of items were: “In general, others treat me as if I am invisible,” “In general, others look through me as if I do not exist,” and “In general, others ignore me during conversation.” The response items ranged from *Never* (1), (2), (3), (4), to *Always* (5). The Cronbach’s α reliability was 0.85.

##### Daily discrimination

To measure discrimination in the everyday life, we used the shortened version of the Everyday Discrimination Scale (Williams et al., 1997). The shortened scale comprises five items and measures how often things have happened to the participants in their day-to-day life, showing good predictive validity of various mental health measures in previous studies (Sternthal et al., 2011). Examples of items were: “You are treated with less courtesy or respect than other people,” “You receive poorer service than other people at restaurants or stores,” and “People act as if they think you are not smart.” Response items ranged from *Never* (1), *Less than once a year* (2), *A few times a year* (3), *A few times a month* (4), *At least once a week* (5) to *Always* (6). The Cronbach’s α reliability for this measure was 0.77.

#### Need-Threat

The original scale was created to gauge needs for *belonging*, *self-esteem*, *meaningful existence*, and *control* after a Cyberball game designed to induce ostracism (Jamieson et al., 2010). For this study, we adapted the measure by altering items referring to game-playing. For example, the item “I felt the other players decided everything” was re-phrased to “I have felt that other people decide everything.” Each subscale included five questions, with response items ranging from *Disagree strongly* (1), *Disagree a little* (2), *Neither agree or disagree* (3), *Agree a little* (4), to *Agree strongly* (5). Examples of items for the belonging subscale were “I feel disconnected,” “I feel rejected”, and “I feel like an outsider.” Examples of items for the self-esteem subscale were “I feel good about myself,” “My self-esteem is high,” and “I feel liked.” Examples of items for the meaningful existence subscale were “I feel invisible,” “I feel meaningless,” and “I feel non-existent.” Examples of items for the control subscale were “I feel powerful,” “I feel I have control,” and “I feel I have the ability to significantly alter events.” Because of the abundance of studies showing that ostracism threatens all of these needs and results in lower levels of control, belonging, self-esteem and a sense of meaninglessness (e.g., Williams et al., 2000; Eisenberger et al., 2003; Zadro et al., 2004; Carter-Sowell et al., 2008; Lakin et al., 2008), we created a need-threat composite measure with high levels indicating high need-threat. The same approach has been successfully applied in previous studies (Jamieson et al., 2010). The Cronbach’s α reliability for the composite measure was 0.86.

#### Control Variables: Age, Gender, Living Arrangement, and Time Spent Homeless

We controlled for participants’ age, gender, current living arrangement, and years spent being homeless. For the latter measure, the participants reported on months being homeless, and the responses were collated into a variable with the following categories: up to 1 year (32%); 2–3 years (22%); 3–5 years (16%), and 5 years or longer (30%).

#### Analytic Strategy

Cluster analysis using SPSS 24.0 was used to classify participants into different internalizing profiles. To investigate the moderating effects of internalizing profiles on the link between ostracism and need-threat, we employed multicategorical moderation analyses with sequential coding (Hayes and Preacher, 2014) using PROCESS 2.16.3 (Hayes, 2013).

## Results

### Descriptives

Table 1 shows the descriptive statistics for all study variables. Symptoms of internalizing were all intercorrelated and were positively associated with measures of ostracism and need-threat in turn.

**Table 1:** Descriptive statistics for all study variables.

	Pearson Correlations														
	
	*n*	Mean	*SD*	α	(1)		(2)		(3)		(4)		(5)		(6)
(1) Generalized anxiety	114	2.65	0.84	0.74	-										
(2) Depressive symptoms	114	2.77	0.63	0.82	0.65	^∗∗∗^	-								
(3) Social anxiety	113	1.56	0.93	0.89	0.32	^∗∗∗^	0.53	^∗∗∗^	-						
(4) Feeling ignored	113	2.11	1.20	0.85	0.13		0.31	^∗∗∗^	0.28	^∗∗^					
(5) Daily discrimination	113	3.18	1.29	0.77	0.18	^†^	0.42	^∗∗∗^	0.34	^∗∗∗^	0.40	^∗∗∗^			
(6) Need-threat	114	3.27	0.85	0.86	0.53	^∗∗∗^	0.61	^∗∗∗^	0.45	^∗∗∗^	0.46	^∗∗∗^	0.35	^∗∗∗^	0.11


*^†^*p* < 0.10. ^∗∗^*p* < 0.01. ^∗∗∗^*p* < 0.001.*

### Identifying Internalizing Profiles

To identify profiles based on symptoms of internalizing psychopathology, we conducted hierarchical cluster analyses using Ward’s method and squared Euclidean distances with generalized anxiety, depressive symptoms and social anxiety (standardizing the variables beforehand). In order to decide on the final number of clusters, we used the following recommended criteria: (1) reasonable homogeneity of clusters as indicated via Explained Error Sums of Squares (EESS) values, which should ideally be around 67% and not less than 50% (Bergman et al., 2003); (2) preferably no less than 10% cluster coefficient percentage change to the next level (Hair et al., 1998); (3) at least 10 individuals per cluster (Hair et al., 1998), and (4) a theoretically meaningful cluster solution (Bergman et al., 2003; Hair et al., 1998).

Ten cluster solutions were subsequently tested. The EESS-values and the change in percentage of the cluster coefficients to the next level for cluster solutions 3–10 are shown in Table 2. All of the cluster solutions between 5 and 10 could be used (as indicated by the EESS value being above 67%), and all were above the recommended value of 10% in terms of the cluster coefficient percentage change. Only cluster solutions 3 and 4 fulfilled the criteria of comprising more than 10 individuals per cluster, but the theoretical meaningfulness of more than 3 clusters was questionable. The patterns emerging for the higher cluster solutions included the same patterns found in the 3-cluster solution, with the size of the participants varying overall across the clusters. Thus, due to considerations of theoretical meaningfulness and the other criteria being fulfilled, the 3-cluster solution was used as the final choice in further analyses (shown in Table 3). Three distinct profiles of internalizing symptoms emerged, with individuals reporting Low (*n* = 22), Medium (*n* = 62), and High (*n* = 29) symptoms of internalizing psychopathology.

**Table 2:** Explained error sums of squares (EESS-values) and changes in percentage of the cluster coefficient to the next level for the cluster solutions between 3 and 10.

Cluster solution	3	4	5	6	7	8	9	10
EESS (%)	52	46	69	73	76	79	82	83
Coefficient (%)	37	23	17	12	13	13	12	11


**Table 3:** Means (Standard deviations) for the 3-cluster solution using standardized variables.

Clusters	Anxiety	Depressive symptoms	Social anxiety	*N* (Women)
Low internalizing symptoms	-1.00 (0.55)	-1.48 (0.73)	-1.19 (0.43)	22 (2)
Medium internalizing symptoms	0.10 (1.02)	0.11 (0.63)	-0.13 (0.64)	62 (16)
High internalizing symptoms	0.54 (0.66)	0.88 (0.48)	1.18 (0.60)	29 (11)


### Does Internalizing Interact With Ostracism in Predicting Threats to Primary Needs?

#### Testing Regression Assumptions

The data were tested for violations of normality, linearity, and homogeneity of variance (Hayes, 2013). The independence of error assumption, an additional important supposition for OLS regression testing, was assumed fulfilled as participants answered questions independent to each other. Tests of normality indicated that feeling ignored was not normally distributed for the Low Internalizers (Shapiro-Wilk = 90; *df* = 22; *p* = 0.03), whereas need-threat was not normally distributed for the High Internalizers (Shapiro-Wilk = 93; *df* = 29; *p* = 0.07). Nevertheless, only the greatest breaches of normality affect the validity of statistical inference from a regression analysis unless sample sizes are very small (Hayes, 2013). Scatterplots with linear fit lines indicated that both feeling ignored (*R*^2^ = 0.21) and daily discrimination (*R*^2^ = 0.12) were moderately linearly related to need-threat. Tests of homoscedasticity were conducted using a one-way ANOVA with Levene’s test of homogeneity of variance, indicating equal variances across the three internalizing profiles. Based on these results, we assumed that conducting further testing using an OLS framework was appropriate.

#### Moderation Testing

As a second step, we conducted two separate models with the two indicators of ostracism (feeling ignored and daily discrimination, respectively) as predictors, need-threat as outcome, and Internalizing profiles as moderator. Age, gender, current living arrangement and time spent being homeless were controlled for in all analyses, with the predictor variables mean-centered (Aiken and West, 1991). As PROCESS only uses information from participants with complete data, 111 participants were included in the final analyses. Interactions were probed using a simple slopes procedure with 1 SD above and below the mean (Aiken and West, 1991; Hayes, 2013). To compare the Internalizing profiles, sequential coding was employed, comparing Low with Medium (D1) and Medium with High (D2) internalizing profiles (see Hayes and Montoya, 2017). When comparing multicategorical variables with *k* groups, PROCESS constructs *k*-1 variables (termed D1 and D2 in Tables 4, 5), which are added to the model including products necessary to specify the interaction (Hayes and Montoya, 2017). Because there were several control variables, group differences are given in adjusted means (Hayes and Montoya, 2017).

**Table 4:** Feeling ignored × Internalizing predicting need-threat as outcome.

Model	Coefficient	*SE*	*t*	*p*	CI_Low_	CI_High_
**Omnibus test**						
Independent variable						
Feeling ignored	0.25	0.13	1.99	0.05	0.00	0.51
Control variables						
Age	0.00	0.01	0.64	0.52	-0.01	0.02
Gender	-0.08	0.17	-0.49	0.63	-0.42	0.25
Living conditions	0.13	0.05	2.41	0.02	0.02	0.23
Years being homeless	0.02	0.06	0.31	0.75	-0.10	0.13
Pairwise comparisons with sequential coding		
D1^a^ (Low vs. Medium internalizers)	0.73	0.19	3.83	0.00	0.35	1.11
D2^b^ (Medium vs. to High internalizers)	0.42	0.18	2.32	0.02	0.06	0.77
Conditional effects of focal predictor for internalizing profiles		
Low internalizers	0.25	0.13	1.99	0.05	0.00	0.51
Medium internalizers	0.30	0.08	3.82	0.00	0.14	0.45
High internalizers	0.12	0.13	0.97	0.33	-0.13	0.37
**Interactions**						
D1^a^ × Feeling ignored	0.04	0.15	0.29	0.77	-0.25	0.34
D2^b^ × Feeling ignored	-0.18	0.15	-1.18	0.24	-0.47	0.12


*^*a*^Difference in means between the Low compared with Medium internalizing profiles. ^*b*^Difference in means between the Medium compared with High internalizing profiles.*

**Table 5:** Daily discrimination × Internalizing predicting need-threat as outcome.

Model	Coefficient	*SE*	*t*	*p*	CI_Low_	CI_High_
**Omnibus test**						
Independent variable						
Daily discrimination	-0.12	0.15	-0.81	0.42	-0.42	0.17
Control variables						
Age	0.01	0.01	1.48	0.14	-0.00	0.02
Gender	-0.01	0.18	-0.04	0.97	-0.36	0.24
Living conditions	0.11	0.05	2.13	0.04	0.01	0.22
Years being homeless	-0.01	0.06	-0.23	0.82	-0.13	0.10
Pairwise comparisons with sequential coding		
D1^a^ (Low vs. Medium internalizers)	0.95	0.22	4.24	0.00	0.57	1.40
D2^b^ (Medium vs. High internalizers)	0.51	0.18	2.85	0.01	0.16	0.87
Conditional effects of focal predictor for internalizing profiles		
Low internalizers	-0.12	0.15	-0.81	0.42	-0.42	0.17
Medium internalizers	0.22	0.07	2.97	0.00	0.07	0.37
High internalizers	0.09	0.11	0.83	0.41	-0.13	0.31
**Interactions**						
D1^a^ × Daily discrimination	0.34	0.17	2.03	0.05	0.01	0.68
D2^b^ × Daily discrimination	-0.13	0.13	-0.98	0.33	-0.40	0.13


*^*a*^Difference in means between the Low compared with Medium internalizing profiles. ^*b*^Difference in means between the Medium compared with High internalizing profiles.*

#### Feeling Ignored as Predictor

The model with feeling ignored as predictor of need-threat is shown in Table 4 (*R*^2^ = 0.42, *F* = 8.27, *df*_1-2_ = 9–101, *p* = 0.0000). Feeling ignored was a significant predictor of need-threat. Mean differences between Low vs. Medium Internalizers (D1) and between Medium vs. High Internalizers (D2) were also significant predictors of need-threat, and living arrangement was the only covariate with a significant effect in the model. The conditional effects of feeling ignored were significant for Low and Medium Internalizers, indicating variation in slopes across those two levels of internalizing particularly. Nevertheless, the interactions between Low vs. Medium (D1) and Medium vs. High (D2) Internalizing (i.e., differences between the slopes relating to profiles) and feeling ignored were not significant predictors of need-threat.

#### Daily Discrimination as Predictor

**FIGURE 1:**
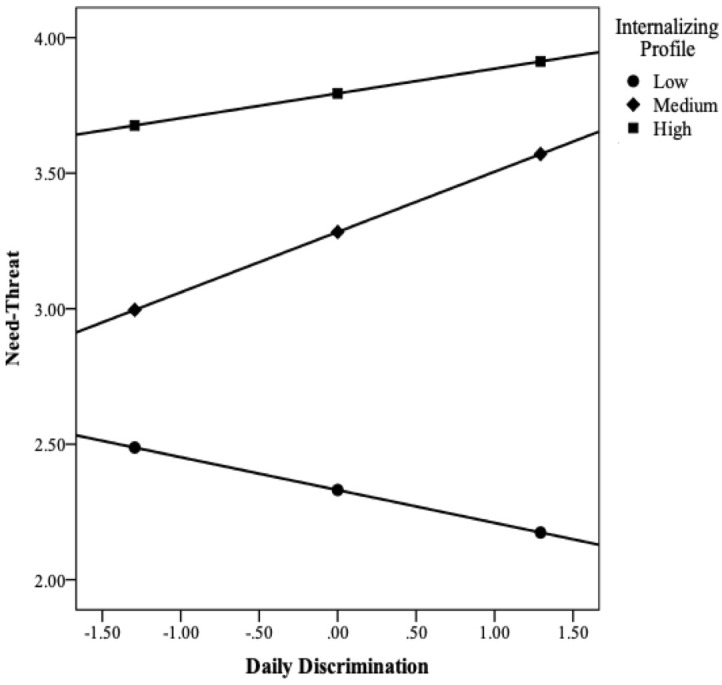
Moderating effects of internalizing on the links between daily discrimination and need-threat (using the simple slopes procedure).

The model with daily discrimination as predictor of need-threat is shown in Table 5 (*R*^2^ = 0.38, *F* = 6.74, *df*_1-2_ = 9–101, *p* = 0.0000). Daily discrimination was not a significant predictor of need-threat. Mean differences between Low vs. Medium Internalizers (D1) and between Medium vs. High Internalizers (D2) were significant predictors of need-threat, as was living arrangement. In addition, the conditional effect of daily discrimination was significant for the Medium Internalizers only. Nevertheless, a significant interaction emerged (*R*^2^_change_ = 0.03; *F* = 2.16; *df*_1_ = 2; *df*_2_ = 101; *p* = 0.12) between Low vs. Medium Internalizing profiles (D1) on the links between daily discrimination and need-threat, which was probed using the simple slopes procedure (Hayes and Montoya, 2017) and plotted in Figure 1.

As can be seen in the figure, and contrary to expectations, Low Internalizers with 1 SD below the mean on daily discrimination had the *highest* level of need-threat, whereas those with 1 SD above the mean on daily discrimination had the *lowest* level of need-threat. For Medium and High Internalizers, however, the pattern was the opposite and as expected – compared to 1 SD above the mean on daily discrimination, 1 SD below the mean was associated with lower levels of need-threat. Nonetheless, the effect of daily discrimination on need-threat was significant for the Medium Internalizing condition only, which could be due to power issues. Taken together, these results indicate that internalizing moderated the link between daily discrimination and need-threat for homeless individuals, with particular effects for those with Medium levels of psychopathology.

## Discussion

Exposure to ostracism and social exclusion is a common experience for homeless individuals (Carpenter-Song et al., 2016; Van Straaten et al., 2018), as is internalizing psychopathology such as depressive symptoms and anxiety (e.g., Edens et al., 2011; Fitzpatrick et al., 2012, 2015; Cruwys et al., 2014, Fazel et al., 2014, Fitzpatrick, 2017). Nevertheless, relatively little attention has been devoted to exploring the impact of existing psychopathology on the links between perceived ostracism and threats to fundamental needs. This study used a person-oriented approach to identify commonly occurring profiles of internalizing problems characterized by symptoms of social anxiety, generalized anxiety, and depression in a sample of homeless individuals with multiple complex needs living in London. We used measures of feeling ignored and daily discrimination to indicate perceived social ostracism, whereas sense of belonging, self-esteem, meaningful existence, and feelings of control were combined to indicate threats to fundamental needs. Controlling for the effects of age, gender, current living arrangement, and time spent being homeless, our results showed no effects of internalizing on the links between feeling ignored and need-threat. Nevertheless, for Medium Internalizers, low levels of daily discrimination were related to high threats to fundamental needs. This pattern was similar for High Internalizers but reverse for Low Internalizers, though the conditional effect of the predictor on need-threat wasn’t significant for these groups.

Though feeling ignored was a significant predictor of ostracism in this study, the results showed no moderating effects of internalizing psychopathology on these links, which could be due to the nature of ostracism. For instance, scholars have argued that ostracism is a “strong situation,” and its immediate effects are not necessarily moderated by individual differences (McDonald and Brent Donnellan, 2012). Even though this explanation is usually associated with instantaneous effects of ostracism, it might still apply to the current study conditions, as our measure of feeling ignored is relatively close to Williams’ proposed ostracism conceptualization and our results might indicate that being ignored had similar associations for participants regardless of internalizing. Similarly, Zadro et al. (2006) found no differences between individuals with varying levels of social anxiety on immediate need-threat scores under conditions of social exclusion – though the participants were not homeless. Ostracism is a process, nonetheless, and interpreting how a self-reported cross-sectional measure of ostracism fits within a proposed temporal model is challenging. It is possible that the measure of feeling ignored forced the study participants to relive their former experiences of rejection, thus producing an effect similar to those observed in immediate measures following an experimental ostracism manipulation. Finally, homeless individuals might feel ignored in such an ongoing manner, that it produces a ceiling effect regarding internalizing psychopathology, but research comparing non-homeless participants with similar internalizing vulnerabilities is needed to test this.

The finding that High and Medium Internalizers experienced greater need-threat related to daily discrimination (with significant conditional effects for Medium Internalizers particularly) could potentially indicate that they ruminate more on these experiences. Such an explanation is consistent with previous findings indicating that rumination – a key component of internalizing – inhibits recovery from ostracism (Wesselmann et al., 2013). High and Medium Internalizers might also have been more susceptible to mental health problems and thus simultaneously identified with two stigmatized groups – homeless and those with mental health problems (Phelan et al., 1997). Interestingly, the pattern of results for Low Internalizers was the opposite (though the conditional effect was non-significant, potentially due to power issues), and the explanation might relate to how such individuals perceive prejudice. For instance, prejudice increases group identification for some individuals, which in turn increases well-being (Schmitt and Branscombe, 2002). This increased identification likely amplifies feelings of belonging and self-esteem, thus lowering threats to fundamental needs. It is worth considering the nature of the two measures of ostracism in the current study. The measure of feeling ignored focuses purely on the individual, whereas the daily discrimination measure asks about comparative experiences. Participants may thus be more able to attribute discrimination externally (i.e., to the person treating them unfairly, or to broader systems of inequality), with Low Internalizers most inclined to make such attributions. On the other hand, recollecting particular instances of feeling ignored could be more difficult, as it might be taken more personally than general discrimination. Future research exploring the basis of perceived discrimination and its impact on fundamental human needs could shed further light on these conceptual issues.

In this study, we tested internalizing psychopathology as a moderator on the links between social ostracism and threats to fundamental needs. Internalizing can function as a lens through which people view the world, thereby creating a risk or a vulnerability; it can be a consequence of experiences and behaviors, or it could very likely be both (i.e., a risk factor *as well as* a consequence). For this study, the aim was to test whether differing patterns of internalizing psychopathology would function as a specific liability in people’s reactions to social ostracism and its links to need-threat. The temporal sequence of internalizing, ostracism and need-threat wasn’t the focus, and we assumed that the homeless participants likely already had existing internalizing problems – making the issue of temporality moot. For example, research on the general population indicates that internalizing psychopathology likely aggravates individual responses to hardships (Keltner and Kring, 1998), which is probable for our chosen sample as well (though this hadn’t been tested before). In addition, when individuals spend time ruminating on their ostracism experiences, which people with internalizing psychopathology commonly do, their ostracism recovery is slower (Wesselmann et al., 2013). Importantly, testing a temporal sequence between internalizing as precursor and/or outcome in these processes is impossible without the use of longitudinal or experimental data – something which future studies should aim to explore further.

Homelessness might be a type of social exclusion that leads to creating stable cognitive models or schemas of social isolation, which in turn increase the risk for developing further mental health problems (Cruwys et al., 2014). One variable we did not measure in the current study, which might nevertheless impact these links, is sensitivity to rejection. For example, participants with experiences of prolonged relational ostracism report that such experiences made them more sensitive to rejection and reluctant to seek out other relationships, which had the result of isolating them further (Zadro et al., 2008). Similar effects have been reported with African-American college students, as those who had experienced status-based rejection where more likely to exhibit rejection sensitivity (Mendoza-Denton et al., 2002). In addition, women high in rejection sensitivity who experienced rejection became more depressed (Ayduk et al., 2011). Experiences of ostracism can thus become self-perpetuating and lead to increased psychopathology. Even though the aforementioned samples include non-homeless individuals, one might expect similar – if not enhanced – links for homeless people. Despite their daily discrimination levels being higher, perhaps individuals with low internalizing patterns have less rejection sensitivity, thereby dampening the effects on need-threat. More research on this topic with a larger number of homeless individuals is necessary in the future.

The current study has several limitations. First, we only have cross-sectional data, thus excluding the possibility of examining the temporal sequence or the stability of these processes over time. Nevertheless, recruiting participants from the homeless population is difficult; but if possible, longitudinal research could track the links between extended periods of homelessness and learned helplessness or exacerbation of internalizing conditions, whereas qualitative studies could investigate how this is experienced by the individual. Second, our measures of ostracism and need-threat are indicators of how the homeless individuals felt on a daily basis, whereas measures of ostracism in the literature have relied on experimentally induced ostracism (e.g., during a game of Cyberball), with levels of perceived need-threat measured afterward. As we did not have the possibility to conduct an experiment with homeless individuals, we adapted this measure into a self-report gauging common daily experiences. In addition, indicators of internalizing psychopathology were measured on different time scales (referring to the past week for symptoms of anxiety and depression, with no time constraints assumed for the measure of social anxiety). Symptoms of anxiety and depression are indeed likely to fluctuate, whereas social anxiety or shyness is considered more of a stable trait (Crozier, 2000). Nevertheless, because all the measures assess different time granularity, this remains a limitation and an empirical challenge for self-reported data estimating such experiences. Third, the variables that were controlled for in the analyses might have important effects in themselves, though due to the sample size and subsequent power issues these were not explored beyond co-variation. Fourth, the use of cluster-analytic techniques prompts a high level of subjectivity regarding decisions about final number of clusters, and some of the clusters were small in size. Nevertheless, this is a limitation shared by all studies relying on individual profiling. Finally, it became apparent during the data collection that it was challenging to gain female participation, resulting in significantly fewer women than men in the final sample. Even though gender was controlled for in the analyses, the need to engage homeless women in future research remains great. Despite its limitations, however, the study has several strengths. First, we have attempted to measure the links between ostracism and need-threat in a sample of homeless individuals with multiple complex needs currently living in London, whereas their age, gender, living conditions and time spent being homeless were controlled for in all analyses. We also adopted a person-oriented approach in identifying profiles of internalizing symptoms to distinguish between profiles of participants and test moderating effects of psychopathology on the links between social ostracism and need-threat. The study thus provides a unique insight into how homeless individuals with varying patterns of internalizing psychopathology perceive ostracism in their everyday life, and the impact this might have on threats to their most fundamental needs as human beings.

In England and other parts of the United Kingdom, homelessness has risen by a staggering 132% since 2010 (e.g., Fitzpatrick et al., 2017). Understanding what factors contribute to a prolonged sense of social isolation is crucial, as is taking into account that homeless individuals likely differ on patterns of mental health problems. An interesting extension of the current research would be to compare a sample of homeless with non-homeless individuals with similar profiles of internalizing psychopathology, as current theories on ostracism predict that homeless individuals are likely to be worse off in terms of threatened fundamental needs (Williams, 2007). Adopting a person-oriented approach in analyzing individual psychopathology patterns not only complements commonly used variable-centered approaches but is vital if we are to understand more about why ostracism has an impact on threats to fundamental needs for some, but not all individuals. More knowledge about this topic would provide a basis for interventions aimed at making homeless people feel more included in their surroundings. Furthermore, understanding why discrimination in particular has different effects on individuals with varying profiles of internalizing psychopathology might help inform strategies for coping with social ostracism more generally.

## Ethics Statement

This study was carried out in accordance with British Psychological Society guidelines. All subjects provided written informed consent in accordance with the Declaration of Helsinki. The study protocol was approved by the University of Greenwich Ethics Review Board.

## Author Contributions

Both authors generated research ideas. NVZ prepared the literature search, conducted the statistical analyses, and wrote the manuscript. RS provided additional literature and comments on subsequent drafts.

## Conflict of Interest Statement

The authors declare that the research was conducted in the absence of any commercial or financial relationships that could be construed as a potential conflict of interest.
